# The role of periostin in tissue remodeling across health and disease

**DOI:** 10.1007/s00018-013-1494-y

**Published:** 2013-10-22

**Authors:** Simon J. Conway, Kenji Izuhara, Yasusei Kudo, Judith Litvin, Roger Markwald, Gaoliang Ouyang, Joseph R. Arron, Cecile T. J. Holweg, Akira Kudo

**Affiliations:** 1 Program in Developmental Biology and Neonatal Medicine, Wells Center for Pediatric Research, Indiana University School of Medicine, Indianapolis, IN USA; 2 Division of Medical Biochemistry, Department of Biomolecular Sciences, Saga Medical School, Saga, Japan; 3 Department of Oral Molecular Pathology, Institute of Health Biosciences, The University of Tokushima Graduate School, Tokushima, Japan; 4 Department of Anatomy and Cell Biology, Temple University School of Medicine, Philadelphia, PA USA; 5 Department of Cell Biology and Anatomy, Medical University of South Carolina, Charleston, SC USA; 6 State Key Laboratory of Cellular Stress Biology, School of Life Sciences, Xiamen University, Xiamen, China; 7 Genentech, 1 DNA Way, South San Francisco, CA USA; 8 Department of Biological Information, Tokyo Institute of Technology, 4259 B-33, Nagatsuta, Midori-ku, Yokohama 226-8501 Japan

**Keywords:** Periostin, Extracellular matrix, Remodeling, Repair

## Abstract

Periostin, also termed osteoblast-specific factor 2, is a matricellular protein with known functions in osteology, tissue repair, oncology, cardiovascular and respiratory systems, and in various inflammatory settings. However, most of the research to date has been conducted in divergent and circumscribed areas meaning that the overall understanding of this intriguing molecule remains fragmented. Here, we integrate the available evidence on periostin expression, its normal role in development, and whether it plays a similar function during pathologic repair, regeneration, and disease in order to bring together the different research fields in which periostin investigations are ongoing. In spite of the seemingly disparate roles of periostin in health and disease, tissue remodeling as a response to insult/injury is emerging as a common functional denominator of this matricellular molecule. Periostin is transiently upregulated during cell fate changes, either physiologic or pathologic. Combining observations from various conditions, a common pattern of events can be suggested, including periostin localization during development, insult and injury, epithelial–mesenchymal transition, extracellular matrix restructuring, and remodeling. We propose mesenchymal remodeling as an overarching role for the matricellular protein periostin, across physiology and disease. Periostin may be seen as an important structural mediator, balancing appropriate versus inappropriate tissue adaption in response to insult/injury.

## Introduction

Periostin, also termed osteoblast-specific factor 2, is a 93.3 kDa-secreted, vitamin K-dependent glutamate-containing matricellular protein, originally isolated from a mouse osteoblast cell line [1, 2]. It is encoded by the *Postn* gene (genebank D13664) in humans, and to date, transforming growth factor beta (TGF-β) 1, 2, and 3, bone morphogenetic proteins (BMP) 2 and 4, vascular endothelial growth factor, connective tissue growth factor 2, vitamin K, valsartan (an angiotensin II antagonist), and interleukin (IL) 3, 4, 6, and 13 have all been reported to induce periostin expression in a cell-specific context [3].

Periostin is assigned to the family of fasciclins based on its homology to fasciclin I (FAS1), initially identified in insects. Proteins that share homology with FAS1 include β ig-h3, stablin I and II, MBP-70, Algal-CAM, periostin, and periostin-like-factor 1 and 2 [1, 2, 4–8]. The four internal repeat regions of periostin share homology with an axon guidance protein FAS1, containing sequences that allow binding of integrins and glycosaminoglycans in vivo [9]. At the N-terminus, periostin has an EMI domain, which is a small cysteine-rich module of ~75 amino acids. The EMI domain was first named after its presence in proteins of the EMILIN family and is associated with other domains, such as C1q, laminin-type EGF-like, FN3, WAP, ZP, or FAS1 [10, 11].

In keeping with periostin’s matricellular role as having regulatory rather than structural functions, periostin can interact with αv-integrins, induce activation of NF-κB/STAT3 [12–14], PI3K/Akt [15], and FAK signaling [16], and modulate expression of multiple downstream genes including: α-smooth muscle actin (αSMA), collagen, fibronectin, aggrecan, sclerostin, chemokines, and TGF-β1 [17–20].

Although periostin has been the target of a multitude of scientific publications since its first identification in 1993 [1, 2], almost all of the research has been conducted in narrowly defined areas. While considerable in-depth molecular knowledge on periostin is evolving in selected fields [21], the overall understanding of this intriguing molecule remains fragmented. As a matricellular protein, periostin has defined functions in osteology, tissue repair, oncology, cardiovascular and respiratory systems, and in various inflammatory settings and diseases. Extensive research has helped to elucidate its mechanism of action or role in many of these, yet there remain several disease states for which its mechanism of action is still unresolved. Emerging data associates periostin with Th2-associated inflammatory diseases, sparking research in several atopic conditions including bronchial asthma. Furthermore, although several different splice variants of periostin have been described [3, 22, 23], their functional implications are as yet not fully understood. Potentially, distinct splice forms may be associated with different functions in various tissues and disease states.

The aim of this review is to (1) integrate the diverse evidence for the role of periostin across health and disease, and (2) identify an overarching mode of action for this pleiotropic matricellular molecule.

## Role of periostin in health and disease

An overarching mode of action is not obviously apparent and has not been described to date in the wide range of tissues and diseases in which periostin has been reported. However, a closer analysis of the associated literature, detailed here, reveals a commonality related to structural remodeling as an upregulated responder to stress/challenge stimuli, regardless of physiology or disease. In this paper, we summarize the available evidence on periostin expression, its normal role in development, and whether it plays a similar function during pathologic repair, regeneration, and disease in order to bring together the disparate research fields in which periostin investigations are ongoing.

### Osteology

Osteoblast-specific factor 2 was identified in 1993 in preosteoblasts and assigned a role in cell adhesion [2]. It was renamed periostin because of high levels of expression in the periosteum; the layer of connective tissue surrounding bone and responsible for intramembranous bone growth required for the increase in bone diameter, which is related to bone strength. Periostin is also highly expressed in the periodontal ligament (PDL) surrounding teeth and responsible for attaching them to the underlying bone [8, 24–27]. The PDL is the conduit for transmission of load to the bony mandible or maxilla and consequently is an important structure required to maintain healthy dentition and bone. In periostin (*Postn*) −/− mice, collagen fibrillogenesis was disrupted in the periosteum and studies on osteoblasts isolated from calvaria of these mice suggest a role in extracellular matrix (ECM) organization as well [28, 29]. It is well recognized that both the bone and the ligament surrounding teeth respond to mechanical stress by remodeling. However, in *Postn* −/− mice, mechanical loading resulted in disorganized collagen matrix formation and an increase in sclerostin mRNA suggesting a sclerostin-mediated decrease in bone mass in these animals. Moreover, bone architecture in response to mechanical stress was restored with anti-sclerostin blocking antibody injections in these animals [19]. Therefore, under normal circumstances, periostin expression results in reduced sclerostin, thereby preserving bone mass and promoting bone remodeling. In the absence of periostin, the increase in sclerostin results in aberrant bone remodeling and a decrease in bone mass. However, as tendons are key in transmitting the force of contraction from muscle to bone, it is possible that in periostin null mice, tendon collagen organization is disrupted, interfering with effective transfer of force contraction from muscle to bone. Bone remodeling is then negatively affected in the absence of adequate loading (force). As the PDL performs an analogous function in teeth as do tendons in bone, findings from the loss of periostin in the knockout mouse in both of these tissues suggest a crucial role for periostin in mechanotransduction and response to mechanical loading and stress.

During embryogenesis and in the neonate, periostin isoforms are expressed in a specific temporal and spatial pattern, suggesting different functions for these variants in bone development and maturation [24]. In adults, periostin is re-expressed during fracture repair or in response to mechanical stress when bone development and remodeling is required [30]. A complete picture of the differential expression of the periostin isoforms is needed to understand the role of the variants in bone development, maturation, and repair. In vitro findings suggest that periostin’s action on bone formation is through an increase in osteoblast proliferation, differentiation, adhesion, and survival [31]. The absence of periostin in knockout mouse models results in growth retardation and dwarfisms, shorter long-bones, and aberrant epiphyseal plate organization [19, 25], suggesting a role for periostin in bone development/remodeling and bone strength. Periostin mediates its effects on bone remodeling specifically by regulating collagen crosslinking and fibrillogenesis by binding to BMP1 via the EMI domain [32], or under conditions of mechanical stress by binding to Notch 1 and impacting osteoblast differentiation and cell death [33, 34]. In pathology, the expression of periostin is observed in fibrous dysplasia, a benign bone disease [35].

### Cutaneous and connective tissue remodeling

Tissue regeneration in response to insult is associated with increased periostin expression [12]. However, this phenomenon is only transient, starting a few days post-injury, with protein levels peaking after 7 days and mRNA levels increasing slightly beforehand. Repetitive strain injuries have been associated with excess collagen deposition around myofibers, cell necrosis, infiltration of inflammatory cells, and increased cytokine expression. In addition, tendon and neural injuries can occur, leading to subsequent chronic inflammatory responses, followed by residual fibrosis [28, 36]. A periostin-like-factor was located in satellite cells and/or myoblasts, which increased in expression with continued task performance, supporting the hypothesis of a role in muscle repair and/or regeneration [37]. Furthermore, periostin has been shown to be expressed at basal levels in healthy human skin but localizes to the extracellular compartment during tissue remodeling involved in wound repair [38]. Recent studies indicate the contribution of periostin toward dermal regeneration and wound healing, suggesting that periostin may promote defect closure by facilitating the activation, differentiation, and contraction of fibroblasts [12, 13, 39].

### Oncology

Periostin overexpression is observed in various types of cancer [40], including thymoma [41], non-small cell lung carcinoma [42], breast cancer [43], pancreatic ductal adenocarcinoma [44], and in ascites from ovarian cancer patients [45]. It is believed to play a role during invasion, angiogenesis, and metastasis, as demonstrated by in vitro and in vivo experiments [40].

Solid tumor cells express high levels of periostin, yet the function of this matricellular protein during non-solid tumorigenesis and progression remains unclear. Periostin has been reported to promote tumor angiogenesis, migration, and metastases [46], and its overexpression has been shown to enhance invasion and anchorage-independent growth and spread in oral squamous-cell carcinoma [47]. Bao et al. [48] demonstrated that a colon cancer cell line with low metastatic potential, transduced to overexpress periostin, displayed accelerated metastatic growth, and that periostin activated the Akt/PKB pathway via the αvβ3 integrin to promote cancer cell survival. Supporting these observations, retrospective analyses of clinical studies have also shown that periostin expression is associated with a trend to metastasize and correlates with angiogenesis in oral, breast, and colon cancers [46, 48–50]. Furthermore, targeting periostin with a modified DNA aptamer, PNDA-3, that is capable of binding to periostin with high affinity and inhibiting its function, markedly antagonized adhesion, migration, and invasion of breast cancer cells both in vitro and in an in vivo orthotopic mouse breast cancer model [51]. Recent findings also suggest that periostin may have a role in sprouting neovascular endothelial tips of disseminated tumor cells, promoting breast cancer cell outgrowth in a tumor-suppressive microenvironment [52].

Periostin is a driver of the epithelial–mesenchymal transition (EMT) and induces expression of MMP-9, MMP-10, and MMP-13, resulting in the degradation of ECM, believed to be crucial for local tumor spread and/or metastasis [53–55]. Furthermore, it is involved in remodeling the tumor microenvironment, which in turn promotes tumor survival, growth, and invasiveness [47]. This has also been described in the pancreatic parenchyma, in which periostin creates a tumor-supportive niche by sustaining fibrogenic stellate cell activity [17, 56], and in esophageal cancer, in which periostin facilitates tumor invasion [57, 58]. Stromal periostin has also been indicated to play a critical role in metastatic colonization [59–61], by regulating the interactions between cancer stem cells and their metastatic niche. Moreover, stromal periostin has recently been reported to enhance cell attachment of clear cell renal cell carcinoma and proliferation of fibroblasts [62]. Periostin may bridge the gap between the metastatic microenvironment and cancer stem cells to promote metastatic spread by augmenting the Wnt signaling pathway [59, 60]. Interestingly, periostin is highly expressed in human bone marrow mesenchymal stem cells and their derived adipocytes, chondrocytes, and osteoblasts. Periostin-overexpressing human mammary epithelial cells acquire part of the multi-lineage differentiation potentials of mesenchymal stem cells and promote tumor growth and metastasis of human breast cancer cell line [63]. These data indicate that periostin is a critical matricellular component in remodeling tissue microenvironment in tumor growth and metastasis.

### Cardiovascular

Periostin is central in cardiovascular differentiation during in utero development of the cardiac valves and fibrous heart skeleton, and is re-expressed following myocardial injury. In detail, it promotes cardiac mesenchymal stem cell differentiation into fibrogenic lineages, is inhibitory to non‐fibrogenic differentiation, and supports early valvulogenesis [18]. During neonatal remodeling, peak expression of periostin will induce collagen production, compaction, and fibroblast proliferation, mediating increased ventricular wall stiffness and valve functional maturation. In *Postn* −/− mice, postnatal valve leaflets are truncated, interspersed with ectopic cardiomyocytes and smooth muscle, show impaired ECM composition, and exhibit reduced TGF-β signaling [64]. Additionally, periostin is robustly expressed during annulus fibrosus development and abnormalities of this differentiation process may underlie development of certain forms of re-entrant atrioventricular tachycardia [65]. However, periostin is downregulated in the postnatal cardiac fibroblast lineage and remains at a low level of expression, but can be rapidly upregulated within cardiac fibroblast/myofibroblasts in response to insult/injury. It is robustly increased following pressure overload-induced left ventricular hypertrophy, and in turn downregulated after left ventricular hypertrophy regression in both animal and human models [66]. Similarly, periostin was markedly upregulated in mouse models of hypertrophic cardiomyopathy associated with non-myocyte proliferation and fibrosis. Abrogating periostin or TGF-β reduced or extinguished both proliferation and fibrosis and improved heart function [67].

In adult pathologic remodeling following cardiac injury or hypertension, periostin serum levels increase and are linked to accelerated mobilization, tissue engraftment, and differentiation of bone marrow cells into cardiac fibroblasts [68]. Additionally, genetic manipulation of *Postn* within the mouse has demonstrated that periostin itself within the heart does not affect myocyte content and cell cycle activity, but may facilitate scarless healing [69]. As a consequence, *Postn* −/− mice are more prone to ventricular rupture within the first 10 days after myocardial infarction [22], yet survivors showed less fibrosis and better ventricular performance. Furthermore, inducible periostin overexpression protected mice from rupture following myocardial infarction but induced spontaneous hypertrophy with aging [70]. Periostin deposition has also been demonstrated to be involved in repair after vascular injury [71], and there is evidence that periostin insufficiency may contribute to valvular heart disease [3, 72], heart failure [66, 73], and atherosclerosis [74]. Elevated periostin in both normal and pathologic hearts is confined to the cardiac fibroblast (non‐cardiomyocyte) lineages, with TGF-β2 being required for periostin expression [75]. Thus, *Postn* is currently being discussed as a potential target for prevention of heart failure [66, 73].

### Allergic and respiratory diseases

Periostin has been reported to play a role in neonatal lung remodeling. Prolonged hyperoxic lung injury was shown to upregulate periostin, stimulating ectopic accumulation of myofibroblasts expressing αSMA, and leading to alveolar simplification [76]. Indeed, periostin expression is tightly correlated with the presence of αSMA-myofibroblasts, and its dysregulation may be a sensitive indicator of acutely-inhibited alveolar septation during a crucial window of lung remodeling [77].

It is evident that epithelial damage is commonplace in respiratory disease, be it from allergens or viral or bacterial infection. In the lung, periostin expression decreases following acute injury, but then increases substantially following TGF-β activation and the initiation of repair mechanisms, but this may persist beyond the initial insult. Evidence suggests a close relationship between periostin and fibrogenesis in response to pulmonary injury [78].

There is a growing body of evidence regarding the role of periostin in asthma and type 2 inflammatory responses in particular [79–81]. Asthma symptoms in some patients may be exacerbated by chronic inflammation of the airways, largely mediated by type 2 inflammatory cytokines, in particular IL-13, which is produced by a variety of adaptive and innate immune cell types including CD4+ T cells, mast cells, basophils, and the recently described innate Th2 cells (ILC2) [82–85]. IL-13 and IL-4 can stimulate the production of periostin via activation of signal transducer and activator of transcription-6 (STAT6) [79, 80, 86]. Periostin expression is elevated in the bronchial epithelial cells of a subset of patients with asthma and is secreted basolaterally [79, 86]. Periostin localizes to the basement membrane zone and the mesenchymal tissue compartment in the lung and colocalizes with other ECM proteins such as collagen, fibronectin, and tenascin-C [78]. Periostin secreted by airway epithelial cells is able to activate TGF-β-mediated increases in type I collagen production in fibroblasts [86]. Periostin can facilitate the infiltration of eosinophils into sites of type 2 inflammation [87] and modulate IL-13 and IL-5-stimulated eosinophil adhesion and motility, suggesting that periostin may function as a haptotactic stimulus able to guide eosinophils to areas of high periostin density in the asthmatic airway [88], which may contribute to sustained eosinophil-mediated inflammation and fibrosis.

Persistent upregulation of periostin in the airway epithelium is likely to contribute to mechanisms of increased airway fibrosis and decreased airway distensibility [86]. Indeed, expression of periostin in airway epithelial cell brushings strongly correlates with subepithelial fibrosis in asthma [86]. The role of the type 2 inflammatory response and IL-13 in subepithelial fibrosis of bronchial asthma is also well established [89–92], and this has been reported to involve periostin as a downstream component, possibly by its binding to other ECM proteins [78]. The functional implications of elevated periostin have recently been investigated. In a Phase II clinical study of subjects with uncontrolled asthma, despite inhaled corticosteroids (ICS), it was demonstrated that periostin status predicted the response to an anti-IL-13 monoclonal antibody, lebrikizumab. Corren et al. [81] reported that lebrikizumab significantly improved lung function at 12 weeks, and that patients with high pretreatment levels of serum periostin had greater improvement in lung function than did patients with low periostin levels. In a different study (not involving lebrikizumab), following assessment of 224 asthmatic patients treated with ICS for at least 4 years, Kanemitsu et al. [93] reported that high serum periostin was one factor associated with an accelerated decline in FEV_1_. Polymorphisms of the *POSTN* gene were associated with both raised serum periostin levels and a decline in FEV_1_ ≥ 30 mL/year, indicating that these may be useful to identify patients at risk of functional decline.

Furthermore, periostin has been linked with development of fibrosis in the pathogenesis of idiopathic interstitial pneumonia, and idiopathic pulmonary fibrosis (IPF) [94]. It is highly expressed in the lungs and serum of IPF patients in whom systemic periostin levels are inversely correlated with pulmonary function [95]. It has been suggested that periostin acts as an inducer of chemokines in the inflammatory response pivotal for the process of pulmonary fibrosis [20].

In addition, periostin has been implicated in atopic conditions such as dermatitis [14, 96] and rhinitis/rhinosinusitis [97]. In allergic skin inflammation, periostin induction after an initial injury contributes to the establishment of sustained chronic inflammation and tissue remodeling [14]. In chronic rhinosinusitis inflammation is mediated by the matricellular proteins periostin and osteopontin, leading to a proliferative response within the ECM framework and largely remodeling of the sinus histopathology [97].

### Miscellaneous inflammatory diseases

Increased tissue periostin has been associated with several inflammatory conditions, in the fields of eosinophilia (e.g., otitis media [98], eosinophilic esophagitis [87]), ophthalmology (e.g., proliferative diabetic retinopathy [99]), hematology (e.g., bone marrow fibrosis [100]), and fibrotic remodeling (e.g., immunoglobulin G4-related sclerosing sialadenitis [101] and scleroderma [102]).

## Conclusions

In spite of the multiple roles of periostin in health and disease (Table 1), tissue remodeling as a response to insult/injury is emerging as a common functional denominator of this matricellular molecule. Periostin is transiently upregulated during cell fate changes, either physiologic or pathologic. Combining observations across a vast expanse of molecular, biological and clinical areas of research, a common pattern of events may be suggested, including periostin localization into the area of development/insult, EMT, ECM restructuring, and eventually remodeling. Assessing the role of periostin by event rather than by disease suggests that any insult/injury such as inflammation, fibrosis, or EMT may be associated with a marked elevation of periostin levels, regardless of the target tissue or type of stimulus.

**Table 1:** Role of periostin in health and disease

Tissue/disease	Health	Disease/repair	References
Osteology	Intramembranous bone growth, bone development, collagen matrix formation and mechanotransduction	Fracture repair or response to mechanical stress	[19, 29, 30]
Cutaneous and connective tissue remodeling	Unknown	Muscle repair/regeneration, wound healing	[13, 37, 38]
Oncology	NA	Promote tumor angiogenesis, migration and metastases, remodeling tumor microenvironment	[46, 47, 53–55]
Cardiovascular	In utero development	Response to pressure overload-induced left ventricular hypertrophy, repair/remodeling following myocardial infarction, repair after vascular injury	[18, 22, 68, 71]
Allergic and respiratory diseases	Neonatal lung remodeling	Increased airway fibrosis, Th2-driven asthma, and ECM protein binding	[76, 78–81, 87]
Other inflammatory diseases	NA	Proliferation within the ECM framework	[97]

*NA* Not applicable

**Fig. 1:**
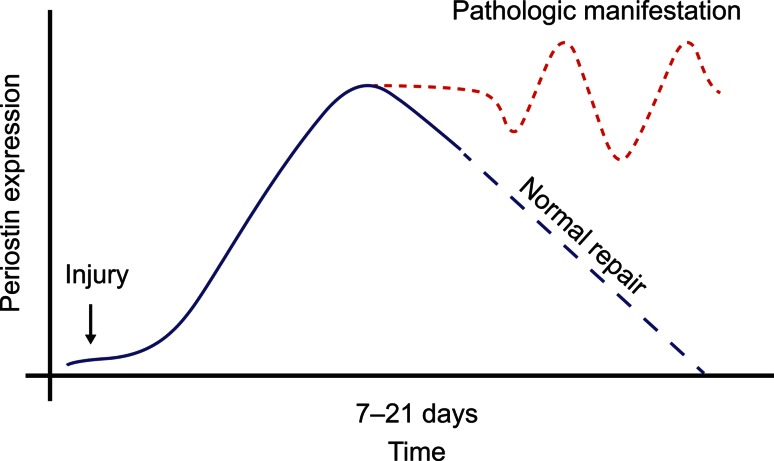
Proposed schematic for transition of periostin’s role in repair to pathologic conditions

There is evidence that a periostin-rich microenvironment develops in areas associated with insult, such as injury and/or inflammation, orchestrating pathways of repair and rebuilding [38, 78]. Exposure to allergens in atopic diseases can be thought of as an insult, similar to what occurs in other inflammatory conditions, in which periostin expression is associated with remodeling, particularly fibrosis and ECM degradation. However, in the presence of inappropriately high and/or persisting periostin upregulation in the absence of an insult, an overshoot of the normal transient repair process can develop (Fig. 1).

**Fig. 2:**
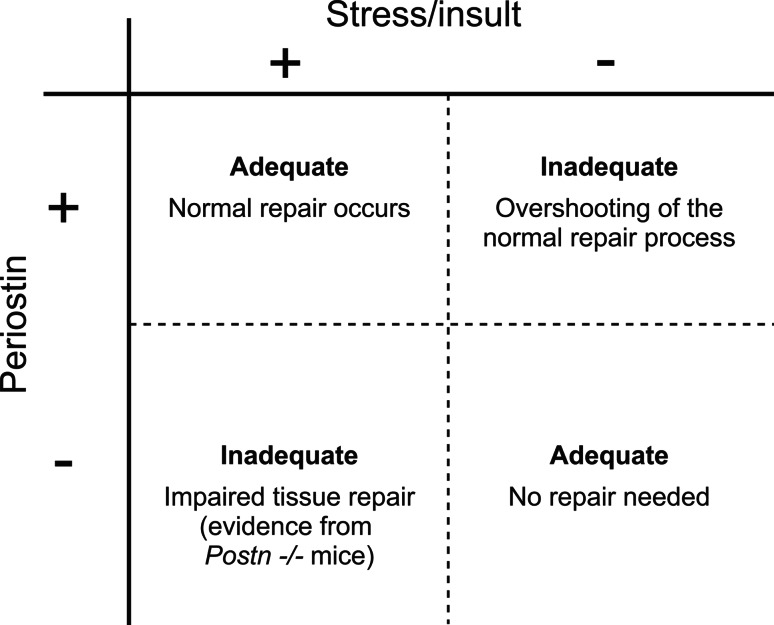
Algorithm for role of periostin in response to stress

Here, an algorithm may be hypothesized, where the appropriate response toward stress/insult is met by a transient periostin upregulation in the targeted tissue/organ (Fig. 2). If periostin expression is exhausted and/or not adequate, the tissue/organ may fail to remodel appropriately, leading to an insufficient response (e.g., mice with cardiac hypertrophy [70]). In contrast, a sustained upregulation of periostin, such as due to a recurring stimulus, could drive remodeling beyond the physiologic adaption and perpetuate, by itself, the disease state (e.g., mice with chronic skin inflammation [14].

Taken together, we propose mesenchymal remodeling as an overarching role for the matricellular protein periostin, across physiology and disease. Periostin may be seen as an important structural mediator in this remodeling process, balancing appropriate versus inappropriate tissue adaption in response to insult/injury.
